# Data-based investigation on the performance of an independent gas turbine for electricity generation using real power measurements and other closely related parameters

**DOI:** 10.1016/j.dib.2019.104444

**Published:** 2019-08-28

**Authors:** Ayodele Benjamin Esan, Vincent Ehiaguina, Claudius Awosope, Lanre Olatomiwa, Dickson Egbune

**Affiliations:** a Landmark University, Omu-Aran, Kwara State, Nigeria; b Covenant University, Ota, Ogun State, Nigeria; c Federal University of Technology, Minna, Nigeria

**Keywords:** Gas turbines, Electrical energy, Energy efficiency, Data analytics

## Abstract

Generally, sub-Saharan countries possess abundant energy resources including renewables and fossil sources, with natural gas potentially being among the more abundant resource second only to solar power. For conventional electrical energy generation, gas turbines are one of the most prominent technologies being adopted in producing electricity from natural gas. Nigeria, for instance has the largest natural gas reserves in Africa, and the 9th largest in the World. Thus, more than 80% of her electricity generation utilizes gas turbines. To effectively monitor the state of these gas turbines, several sensors are located on the turbines to acquire data in real time. In this data article, we present the acquired data from a 5.68-MW gas turbine installed as an independent power producing unit in a community in Ogun State, Nigeria over a period of six months. Performing various descriptive analysis on the dataset, the real power measurements were taken as the target parameters, and based on a threshold correlation co-efficient of 0.5, only sixteen (16) parameters were shown to be more closely positively correlated with the real power measurements. Thus, any variation in the real power supplied by the gas turbine would have a commensurate effect on any of the other 16 parameters identified, and could thus help in troubleshooting or scheduling maintenance.

**Table:** Specifications Table

Subject	Energy
Specific subject area	Energy Engineering and Power Technology
Type of data	Table Chart Figure
How data were acquired	Turbomach Turbotronic 4 Control System. Made by Solar Turbines Incorporated.
Data format	Raw Analyzed Filtered
Parameters for data collection	Over a period of 24 hours, recorded from H1 to H24, all fifty (50) features/parameters of the gas turbine were recorded only when the turbine was operational. No reading was obtained when the turbine was shut-down due to maintenance.
Description of data collection	The Turbomach Turbotronic 4 monitors remotely the readings of all sensors on the gas turbine. It compiles all records in a comma separated file format (CSV) and categorizes them by Day and Night. Due approval was obtained prior to the usage of this data.
Data source location	Ota, Ogun State Nigeria 6.6778° N, 3.1654° E
Data accessibility	Mendeley Data, DOI: https://doi.org/10.17632/6w3vy3ybhg.3 Direct URL to data: https://data.mendeley.com/datasets/6w3vy3ybhg/3

**Table:** 

**Value of the Data** • This data could help data-scientists seeking for ways to utilize machine learning algorithms in identifying fault or scheduling maintenance in a gas turbine. • Independent Power Producers (IPPs) could utilize these data in understanding key features or areas of the gas turbine contributing most to its reliability and stability. • The data shared are relevant for research in the area of power system control and especially for power engineers in troubleshooting and to facilitate the localization of system dysfunctions in gas turbines. • The data is also relevant for energy researchers in proposing novel techniques to curtail the effects of the ambient temperature surrounding gas turbines so as to increase its efficiency.

## 1 Data

Descriptive findings from the correlation matrix of the entire dataset [1] reveal nineteen features of the total fifty features which portray significant positive correlation metrics with the real power produced by the gas turbine. The real power produced by the gas turbine is taken as the target feature or parameter in this data investigation. Prior studies on the effects of ambient temperature on gas powered plants were performed by Refs. [2], [3]. Results obtained indicates significant reductions in turbine's efficiency and electricity production capacities when ambient temperature increases. Thus, other researchers such as [4], [5], [5], [6] proposed novel cooling strategies for natural gas combined cycle power plants (NGCPP). Although natural gas being used in gas turbines denotes a form of fossil-based energy resource, another way through which these sorts of conventional energy resource are being utilized is in hybrid energy systems in terms of micro/mini grids where the conventional source (in this case gas turbines) combined with other renewable energy resources could be harnessed to consistently supply consumer energy demands [7], [8]. In this dataset, the sample space size for every hour of the six months period (July 1st, 2017 till December 31st, 2017) in which this data was recorded is 4416 [1]. However, due to the data clean-ups conducted on the raw data to remove outliers and eliminate null values (when the turbine was shut-down due to scheduled maintenance or gas constraints), the sample space size became 2946. Hence, each of the 19 related parameters had a total of 2946 observations, and have been divided into five different sets as seen in Table 1, Table 2, Table 3, Table 4, Table 5. Each of these 19 related parameters considered for the analysis are briefly explained below:

1) Compressor T5 average: Air Compressor Average Temperature
2) Compressor inlet air temperature (T1): Temperature of the air entering the compressor
3) Lube oil temperature: Temperature of the Turbine lubricating oil.
4) Ceiling temperature: Temperature of the turbine compartment ceiling.
5) Turbine vibration 2X: Turbine vibration of the X-axis
6) Turbine temperature T5 #1: Thermocouple 1- Exhaust Temperature
7) Turbine temperature T5 #2: Thermocouple 2-Exhaust Temperature
8) Turbine temperature T5 #3: Thermocouple 3- Exhaust Temperature
9) Turbine temperature T5 #4: Thermocouple 4- Exhaust Temperature
10) Turbine temperature T5 #5: Thermocouple 5- Exhaust Temperature
11) Turbine temperature T5 #6: Thermocouple 6- Exhaust Temperature
12) Turbine exhaust temperature T7 (Average): Average Exhaust Temperature
13) Gas fuel flow: Flow rate of the gas consumption
14) Generator L1 winding temperature: Temperature of the phase A winding
15) Generator L2 winding temperature: Temperature of the phase B winding
16) Generator L3 winding temperature: Temperature of the phase C winding
17) Current A: Load Current on Phase A
18) Current B: Load Current on Phase B
19) Current C: Load Current on Phase C

**Table 1:** First set of related parameters.

Features	Mean	SD	Median	MAD	Min	Max	Skew	Kurtosis	SE	IQR
Current A	128.0783	50.45164	124	45.9606	0	284	0.157364	−0.2871033	0.9295205	63
Current B	125.9355	50.57952	121	45.9606	0	288	0.2090694	−0.2641368	0.9318764	63
Current C	127.6718	51.44078	122	47.4432	0	281	0.2248668	−0.3214426	0.9477442	64
Gas fuel flow	696.1174	259.7163	717	155.673	0	1258	−1.248423	1.9464801	4.7850098	212

**Table 2:** Second set of related parameters.

Features	Mean	SD	Median	MAD	Min	Max	Skew	Kurtosis	SE	IQR
Turbine temperature T5 #1	420.9838	91.99611	420	72.6474	2.9	644	−0.5440941	1.211722	1.694935	98
Turbine temperature T5 #2	438.3489	79.62829	426	69.6822	24	893	0.5374985	0.7281078	1.467071	98.75
Turbine temperature T5 #3	439.8568	67.41285	432	62.2692	5.9	682	−0.0500536	1.4635095	1.242013	86
Turbine temperature T5 #4	436.4335	86.69454	423	80.0604	24	840	0.5172683	0.0776127	1.597259	110

**Table 3:** Third set of related parameters.

Features	Mean	SD	Median	MAD	Min	Max	Skew	Kurtosis	SE	IQR
Turbine temperature T5 #5	418.8993	83.106816	407	74.13	0.35	872	0.2118989	0.6158988	1.5311589	107.75
Turbine temperature T5 #6	417.9124	76.906636	406	69.6822	24	646	0.4615251	0.0591773	1.4169269	97
Turbine Exhaust temperature T7 (Average)	350.9756	56.906193	339	45.9606	3.2	875	0.9048409	4.6714972	1.048439	67
Turbine vibration 2 X	17.1721	2.718093	17	2.9652	8	33	0.0096114	0.4602953	0.0500781	4

**Table 4:** Fourth set of related parameters.

Features	Mean	SD	Median	MAD	Min	Max	Skew	Kurtosis	SE	IQR
Ceiling temperature	43.96592	3.970685	43	2.9652	4.7	80	0.1536982	10.319183	0.0731559	5
Lube oil temperature	72.46368	4.159743	72	1.4826	4	98	−5.5524509	100.0401	0.0766391	3
Compressor inlet air temp. (T1)	982.08687	435.98801	1169	88.956	0.25	1669	−1.6895842	0.9674669	8.0326377	134
Compressor T5 average	431.52507	79.294473	419.5	70.4235	0.98	1215	0.5272685	3.838034	1.4609204	98.75

**Table 5:** Fifth set of related parameters.

Features	Mean	SD	Median	MAD	Min	Max	Skew	Kurtosis	SE	IQR
Generator L1 winding temperature	57.71154	7.963957	56	5.9304	3.2	84	0.3360594	2.7138029	0.1467278	9
Generator L2 winding temperature	58.48167	7.747578	57	5.9304	24	85	0.7741502	0.5158233	0.1427413	10
Generator L3 winding temperature	58.58239	7.765308	57	5.9304	6.13	84	0.3145802	2.8790363	0.1430679	9

Table 1, Table 2, Table 3, Table 4 present the descriptive statistics of the first four sets of related parameters for the real power produced by the gas turbine, where each set had four independent features respectively. Table 5, however, presented similar descriptive statistics but had only three features. The descriptive statistics considered were the mean, median, median absolute deviation (MAD), skewness, kurtosis, standard error (SE), Interquartile range (IQR), and standard deviation (SD).

**Fig. 1:**
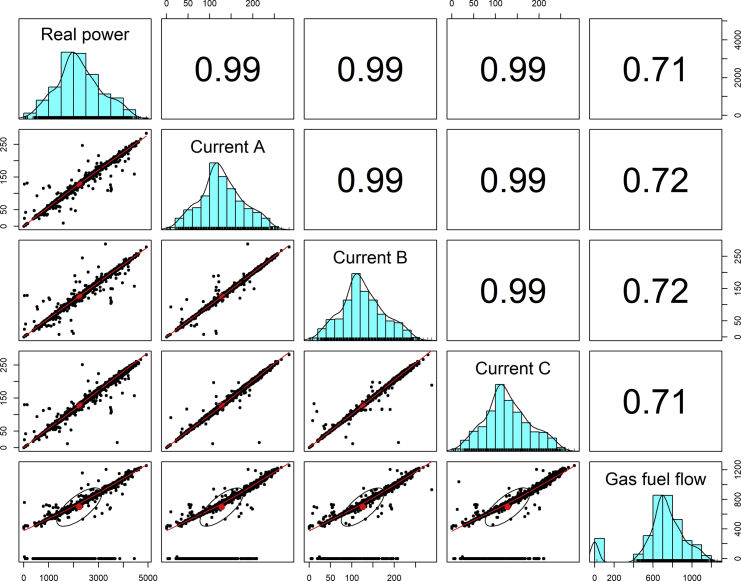
Correlation matrix of first set of related parameters with real power as target.

**Fig. 2:**
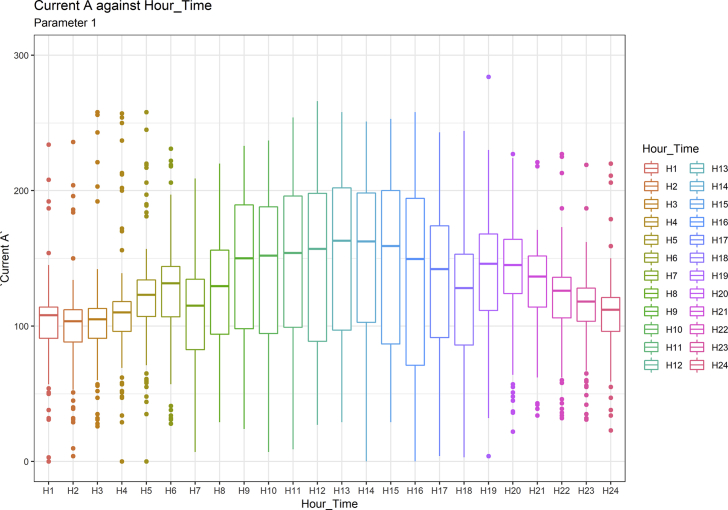
Current in line A by hour time.

**Fig. 3:**
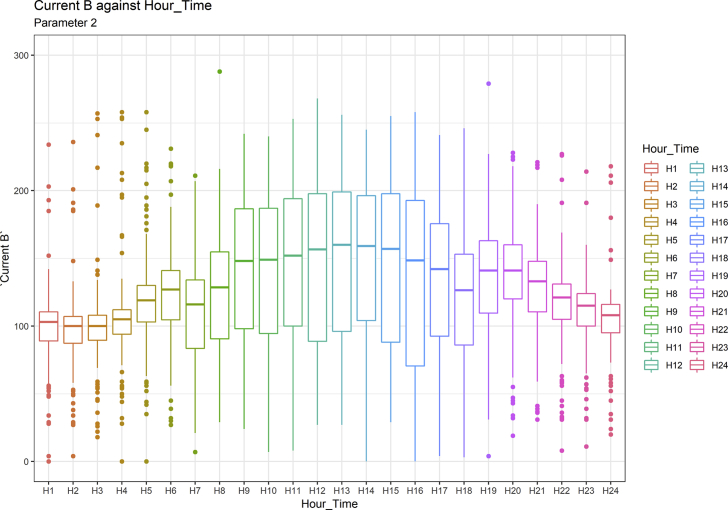
Current in line B by hour time.

**Fig. 4:**
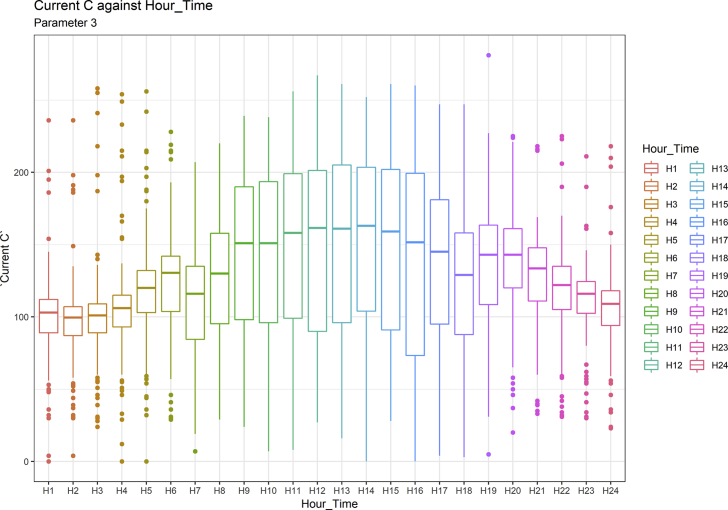
Current in line C by hour time.

**Fig. 5:**
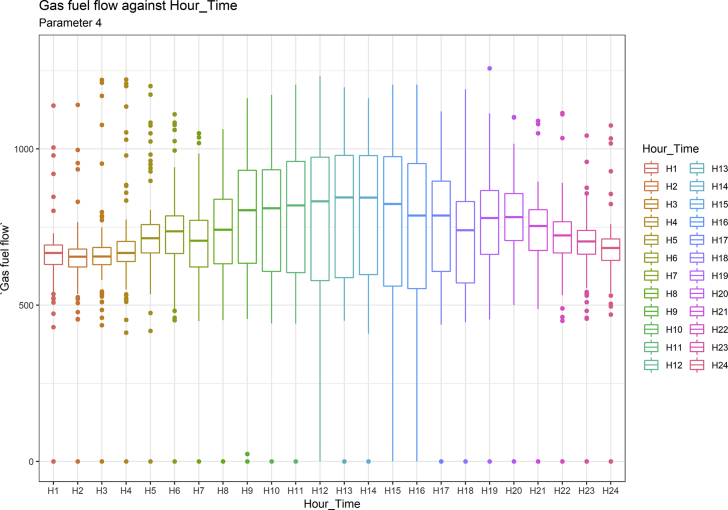
Gas fuel flow by hour time.

**Fig. 6:**
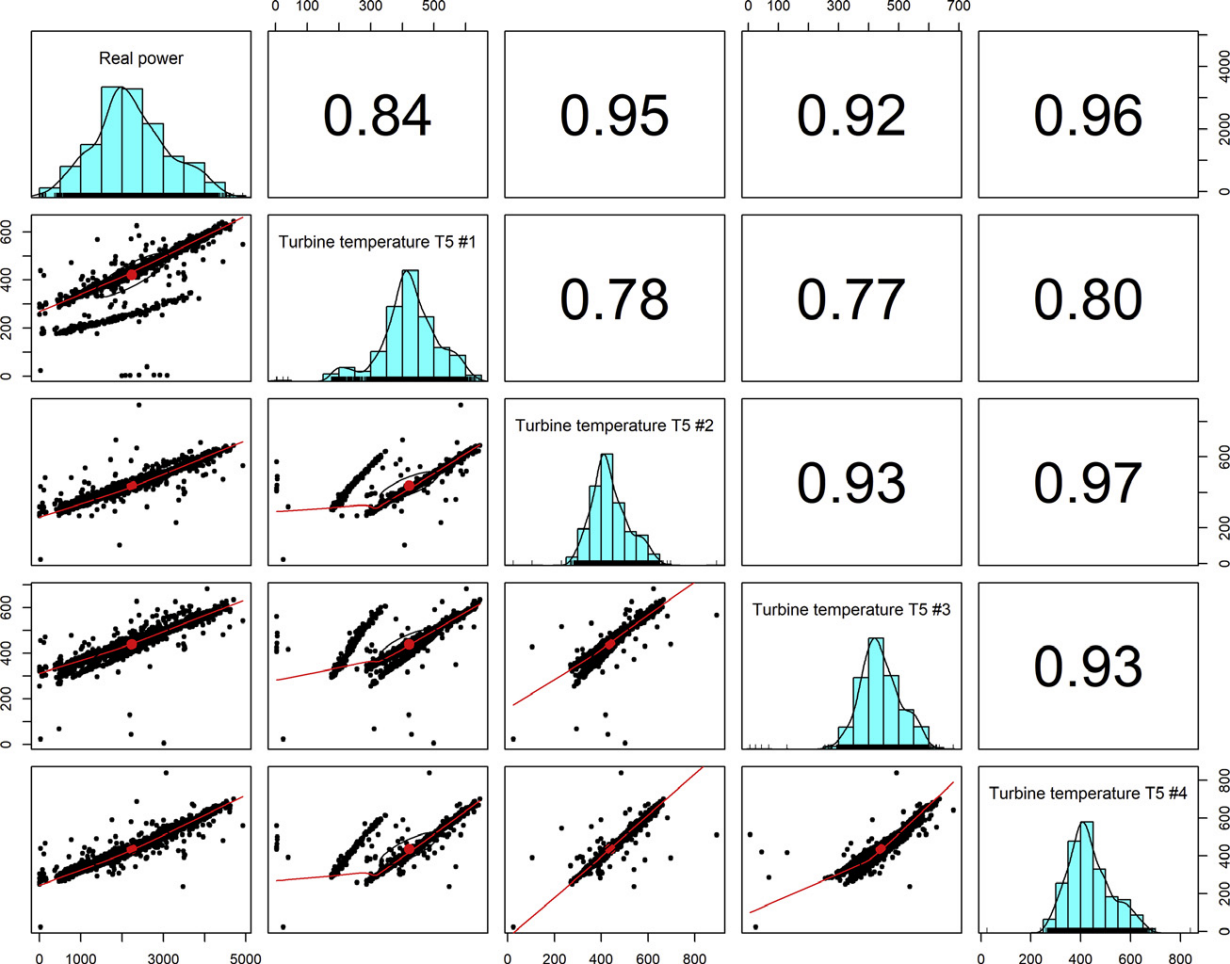
Correlation matrix of second set of related parameters with real power as target.

**Fig. 7:**
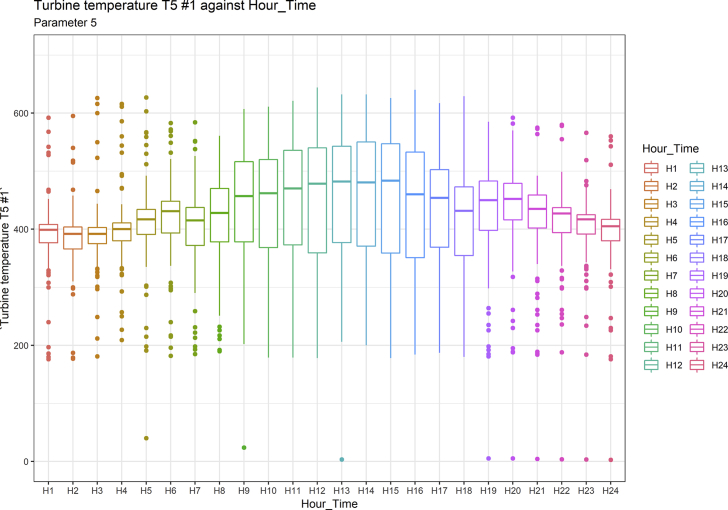
Turbine temperature T5 #1 by hour time.

**Fig. 8:**
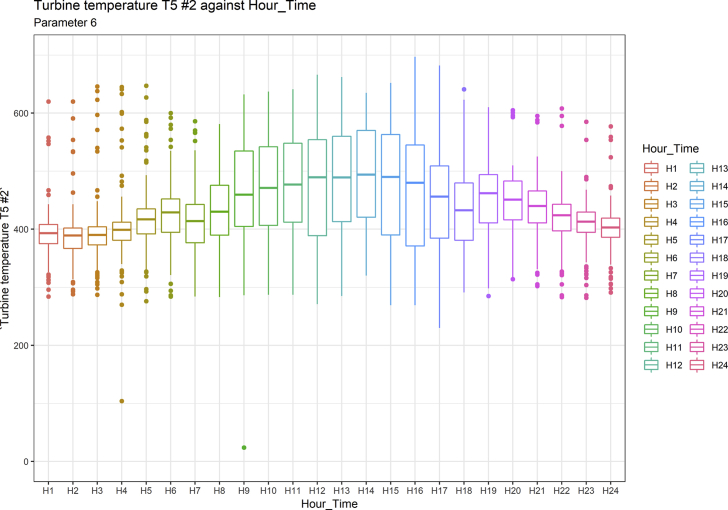
Turbine temperature T5 #2 by hour time.

**Fig. 9:**
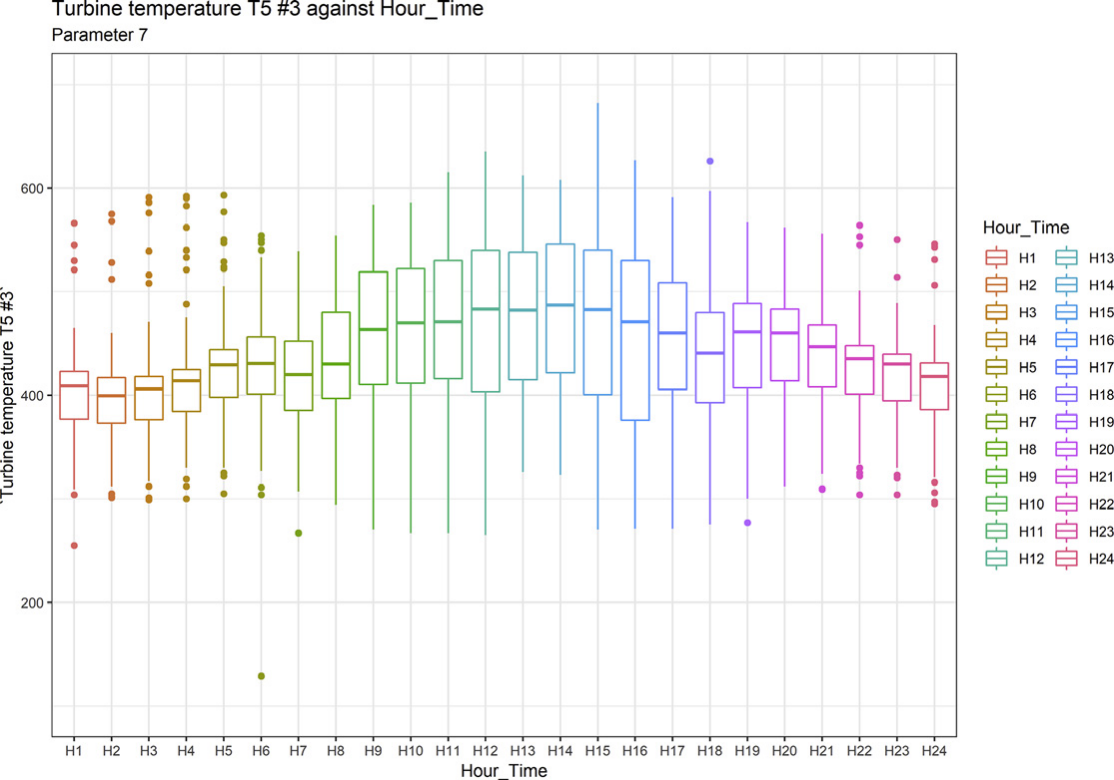
Turbine temperature T5 #3 by hour time.

**Fig. 10:**
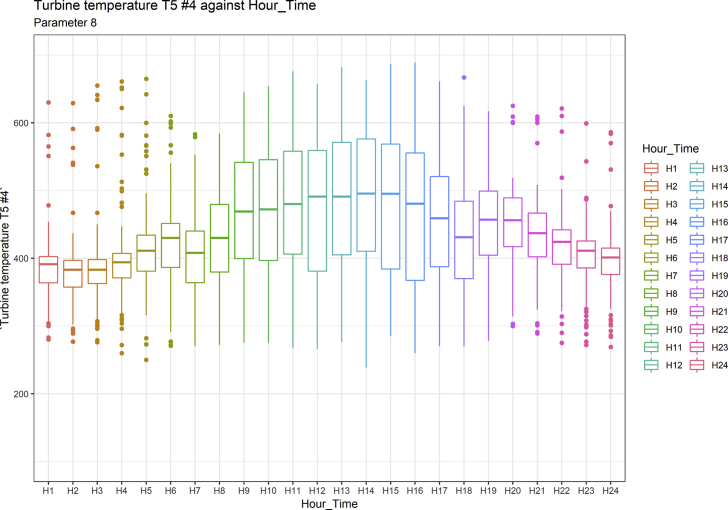
Turbine temperature T5 #4 by hour time.

**Fig. 11:**
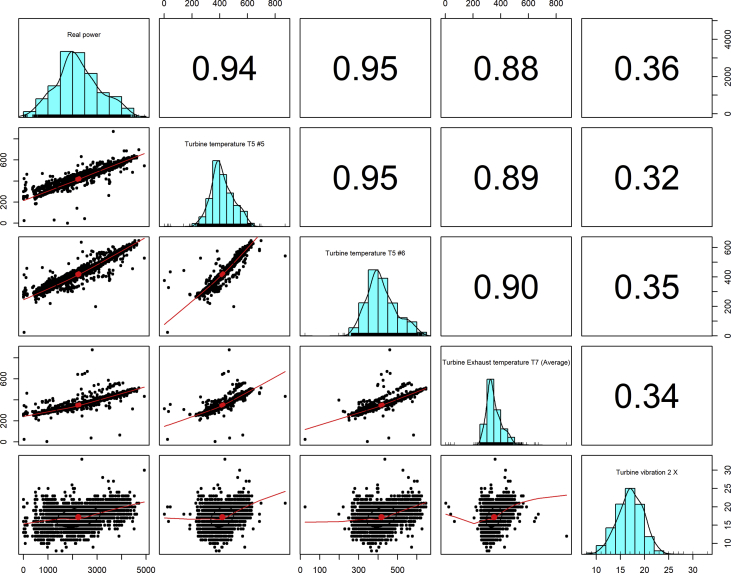
Correlation matrix of third set of related parameters with real power as target.

**Fig. 12:**
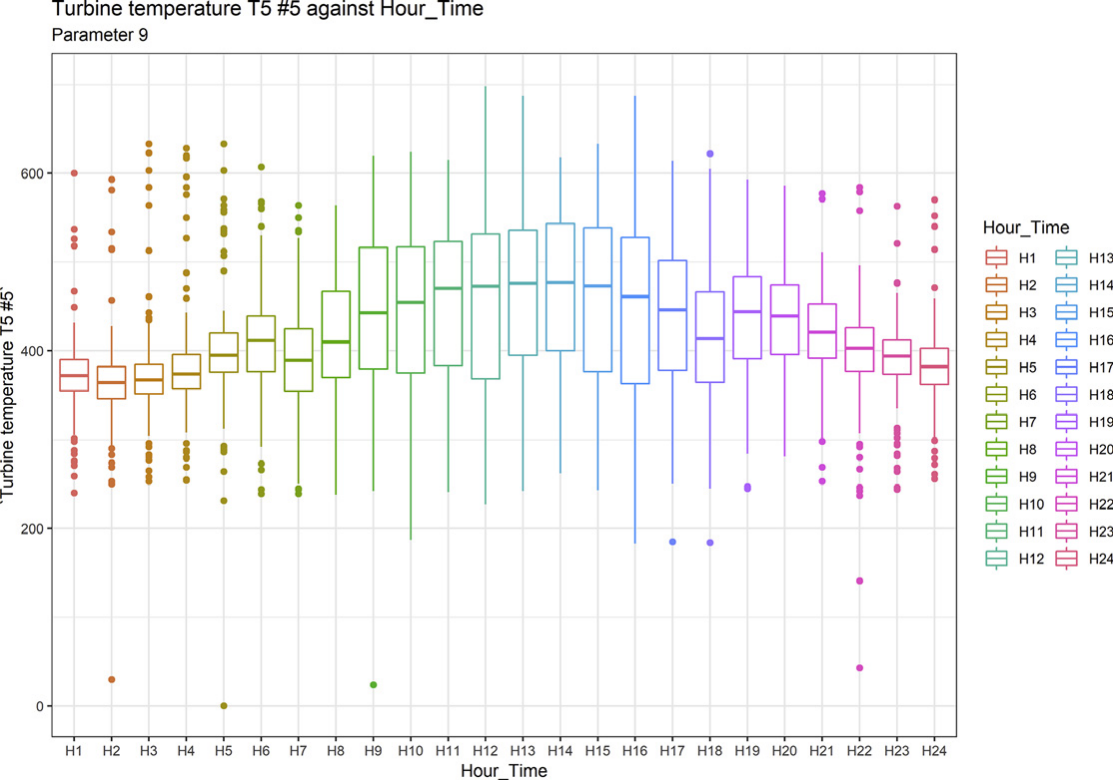
Turbine temperature T5 #5 by hour time.

**Fig. 13:**
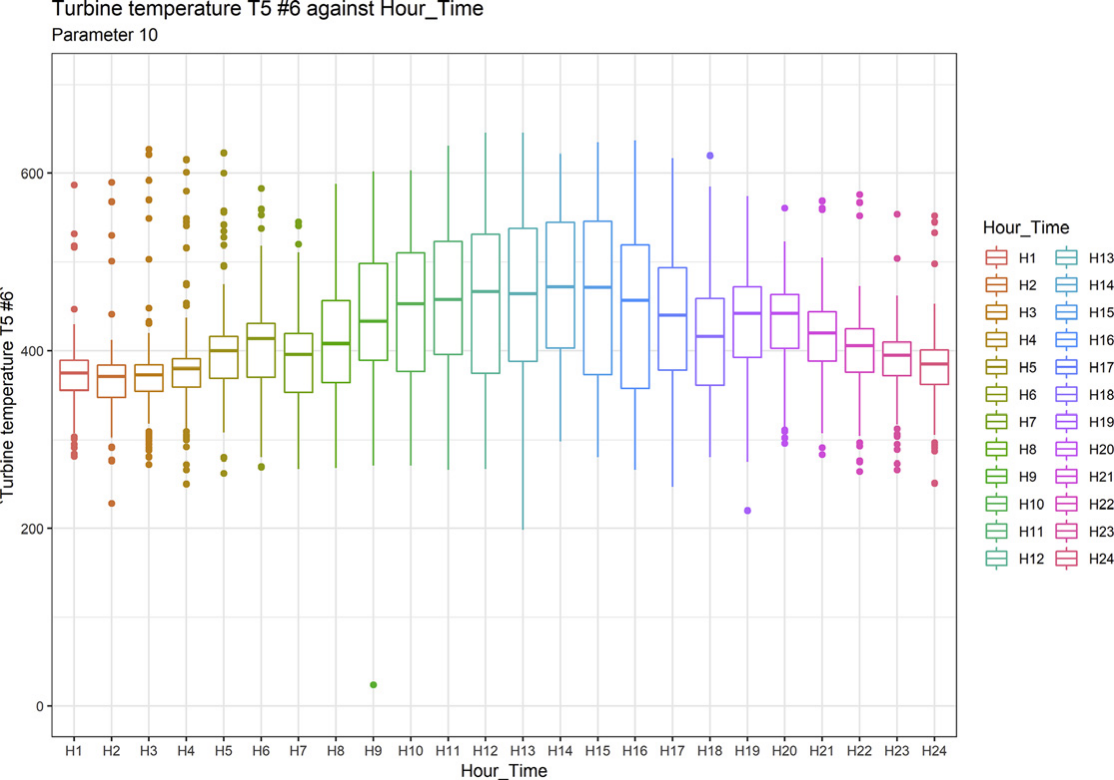
Turbine temperature T5 #6 by hour time.

**Fig. 14:**
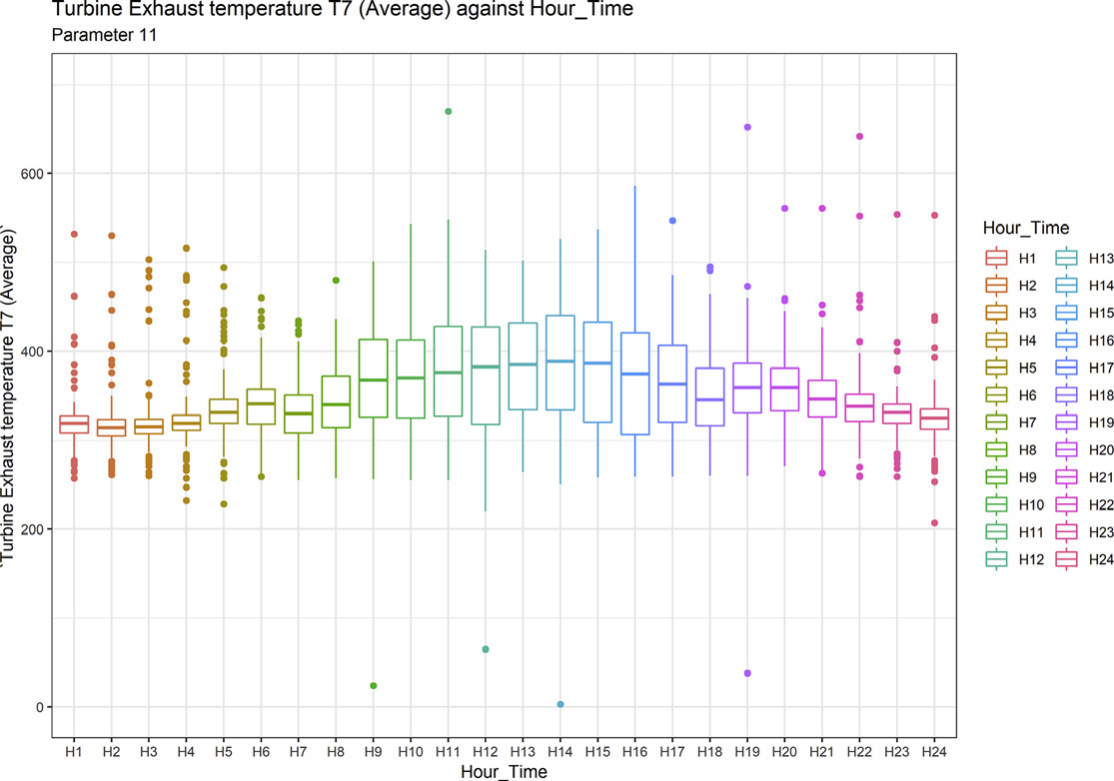
Turbine Exhaust temperature T7 (Average) by hour time.

**Fig. 15:**
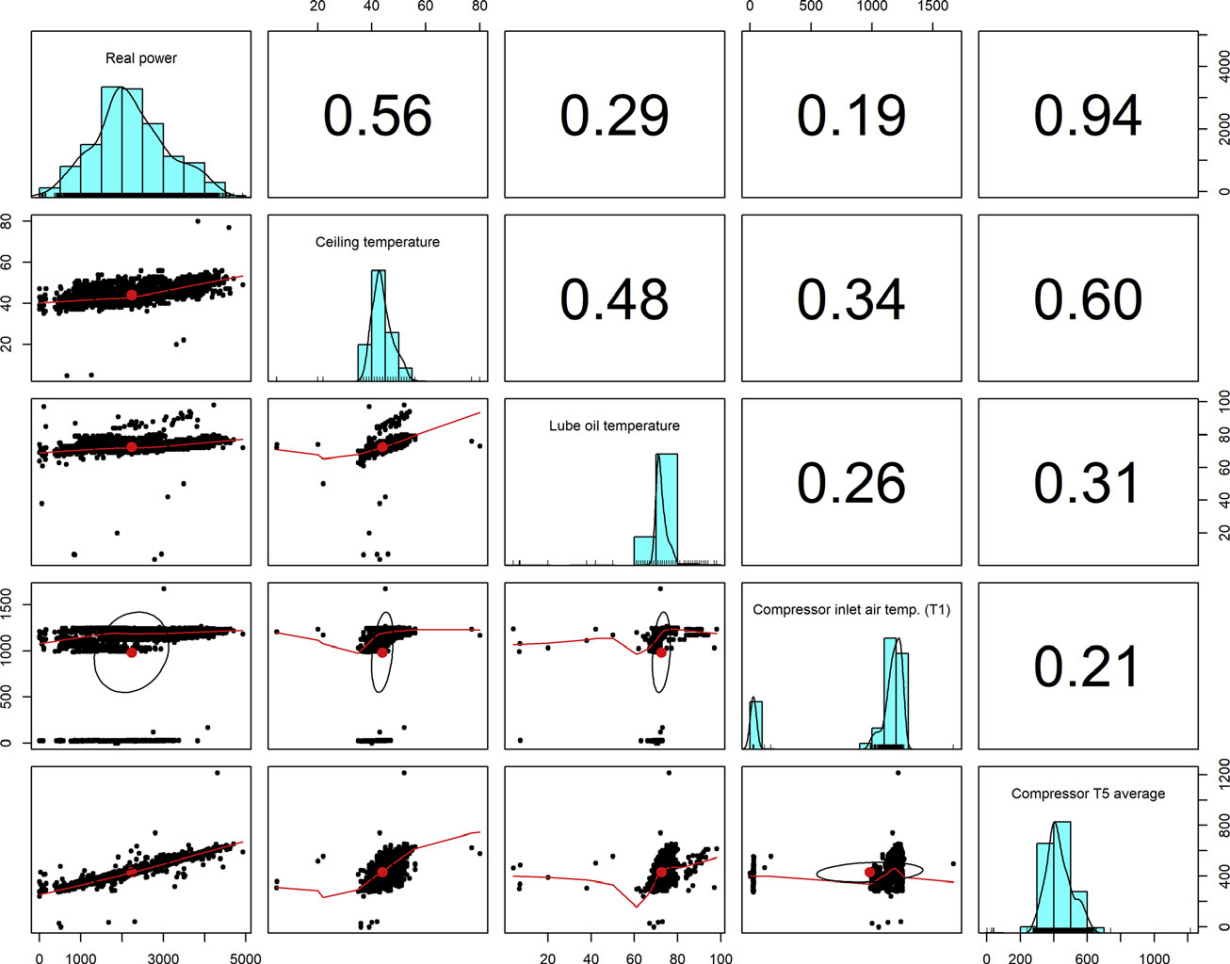
Correlation matrix of fourth set of related parameters with real power as target.

**Fig. 16:**
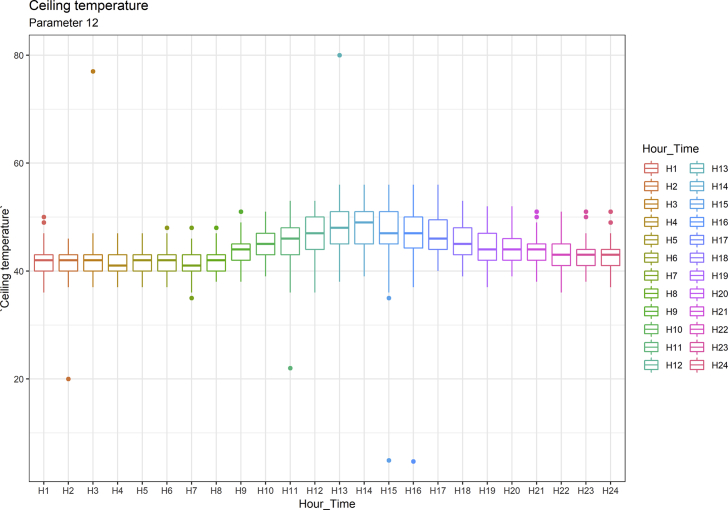
Ceiling temperature by hour time.

**Fig. 17:**
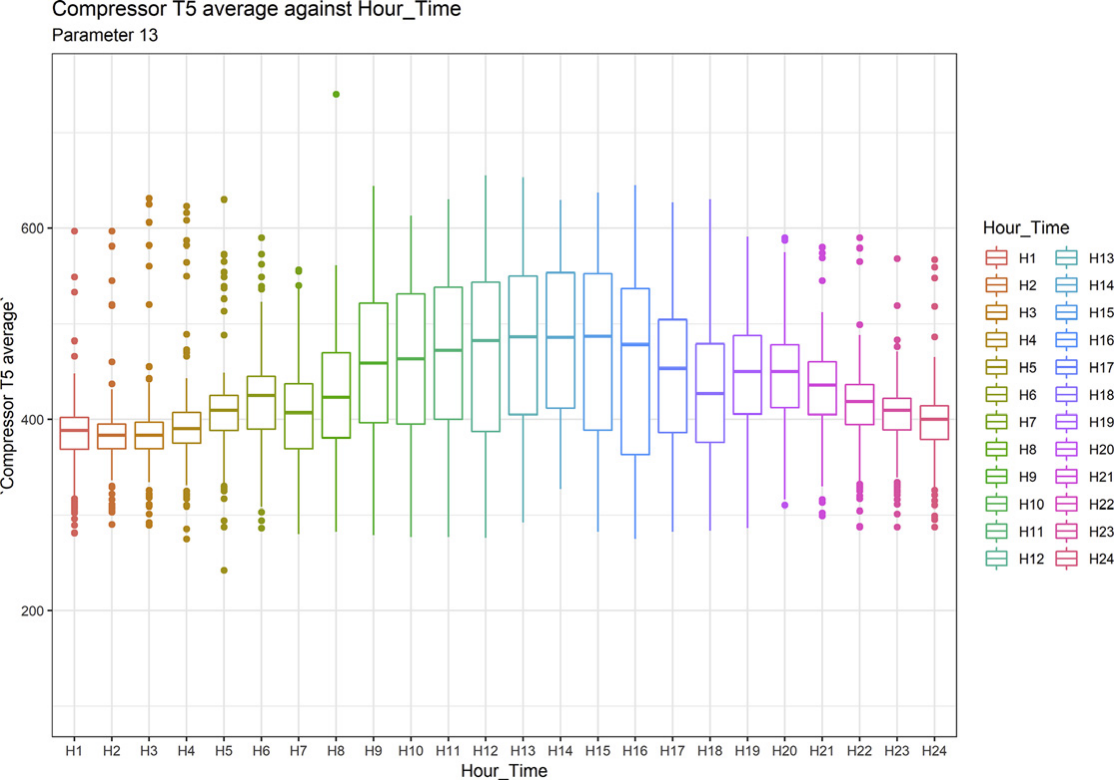
Compressor T5 average by hour time.

**Fig. 18:**
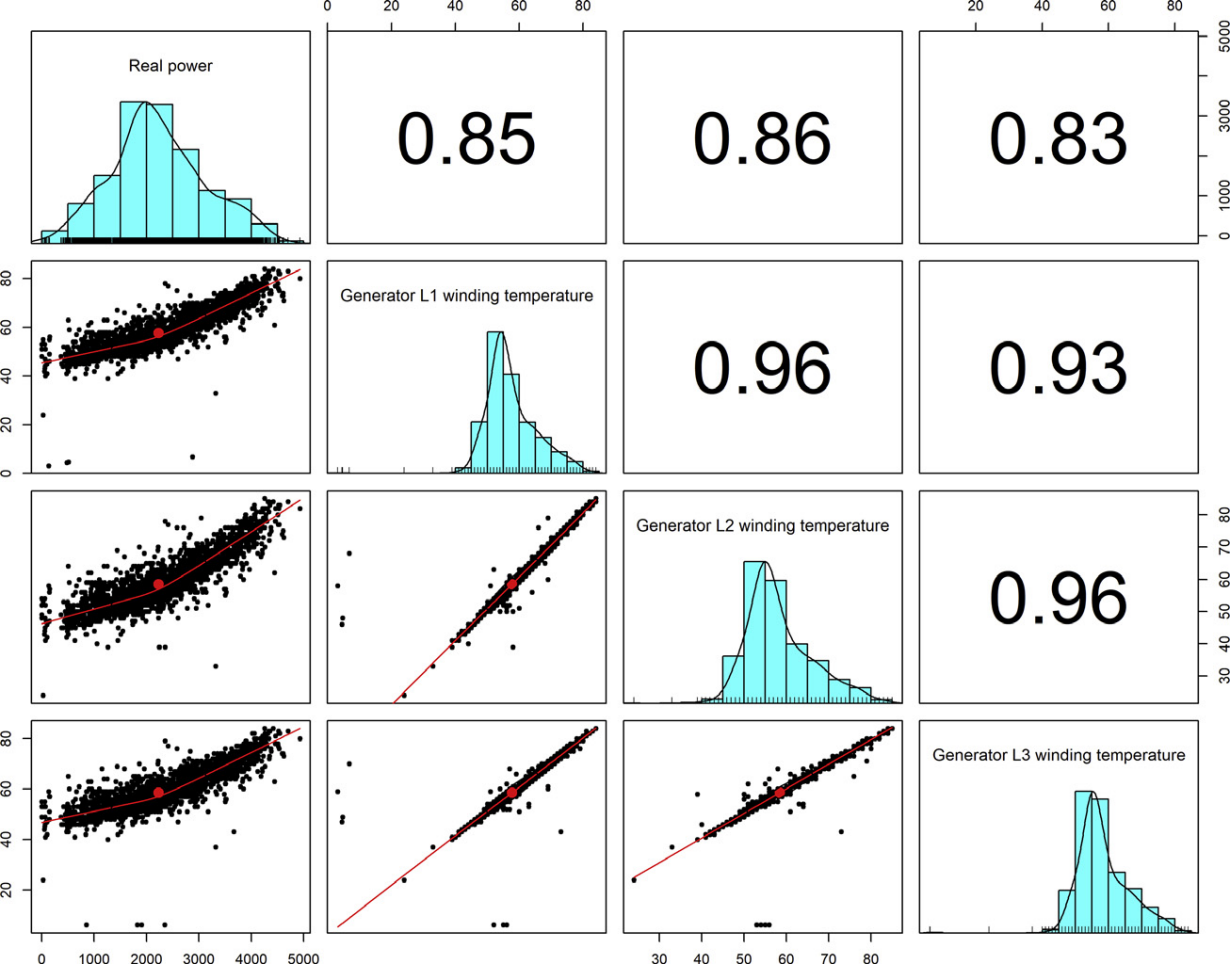
Correlation matrix of fifth set of related parameters with real power as target.

**Fig. 19:**
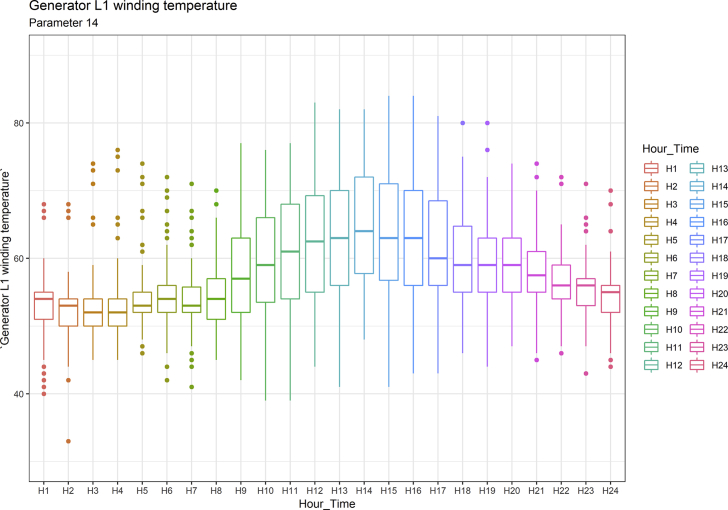
Generator L1 winding temperature by hour time.

**Fig. 20:**
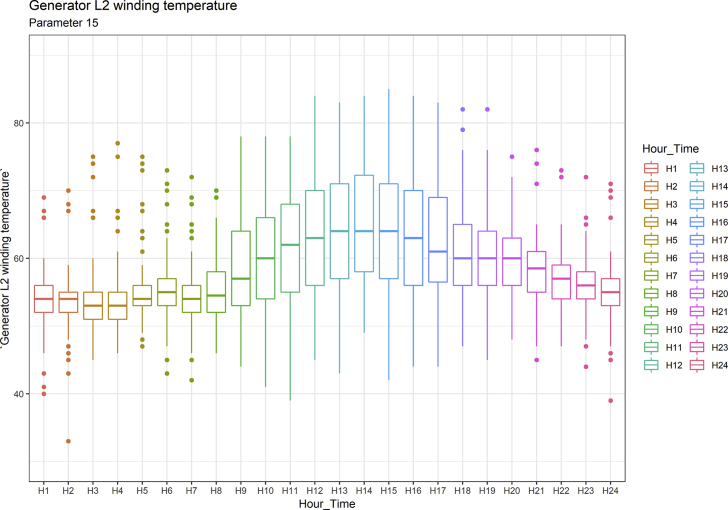
Generator L2 winding temperature by hour time.

**Fig. 21:**
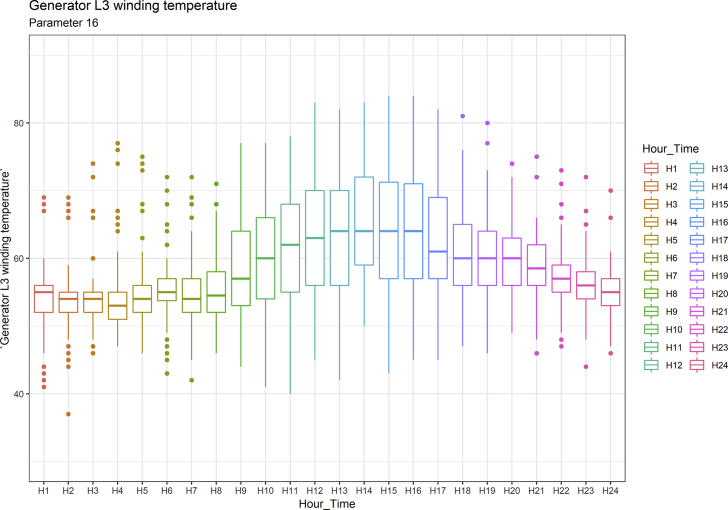
Generator L3 winding temperature by hour time.

Fig. 1, Fig. 6, Fig. 11, Fig. 15, Fig. 18show the graphical correlation matrix of the first, second, third, fourth, and fifth related parameters respectively. A threshold correlation co-efficient of 0.5 was selected in this data investigation. Hence, from the graphical correlation matrix obtained, only correlation co-efficient above 0.5 was selected as having strong correlation with the real power produced. Thus, from Fig. 1, since the correlation co-efficients of all features were greater than 0.5, a boxplot of each feature against the time duration of operation of the gas turbine (H1 to H24) was graphed as shown in Fig. 2, Fig. 3, Fig. 4, Fig. 5. In Fig. 6, all four features also had correlation co-efficients greater than 0.5, hence, Fig. 7, Fig. 8, Fig. 9, Fig. 10 show the boxplot of each of these features against the hour time variable (H1 to H24). Considering the third set of related parameters, from Fig. 11, only three out of the four features considered had correlation co-efficients greater than the threshold value. Hence, Fig. 12, Fig. 13, Fig. 14 show a boxplot of the features against the 24-h time duration of the gas turbine. In the fourth set of related parameters, out of all four features as shown in Fig. 15, only the compressor T5 average parameter and that of the ceiling temperature had a correlation co-efficient greater than 0.5. Hence, Fig. 16, Fig. 17 reveal the boxplots of these parameters against the 24-h time duration of the gas turbine's operation. Lastly, Fig. 18 shows the graphical correlation matrix of the fifth set of related parameters. All three features considered had correlation co-efficients above the threshold value of 0.5. Hence, Fig. 19, Fig. 20, Fig. 21 depict the boxplots of these features against the hour time variable (H1 to H24). As these data was provided by an Independent Gas Turbine Power Plant in Ogun State, the dataset is more representative of most South-Western States in Nigeria due to the relatively similar atmospheric climatic conditions at these locations. It may also prove representative of some regions in sub-Saharan countries like Benin Republic and Togo which possess similar climatic conditions as those experienced in south-western states of Nigeria.

## 2 Experimental design, materials, and methods

The relationships existing between other system parameters/features and the real power measured from the 5.68-MW gas turbine was ascertained using the R statistical programming software (version 3.5.3). A total of 50 features were recorded by the Turbomach Turbotronic 4 SCADA application running on a core i5 2.40 GHz workstation with 1TB of hard-disk space and 4GB RAM. The Turbomach Turbotronic 4 application monitored the gas turbine in real time over a 24-h time period (H1 to H24) and at times when the turbine was shut down due to gas constraints or scheduled maintenance, no values were recorded for the total 50 parameters monitored. The data was recorded by various temperature and pressure sensors installed at various points on the turbine during operation. The data recorded by each sensor changed with every change in load demand and ambient temperature. A transmitter transfers the measured data to a remote Human-Machine Interface (HMI) in the control room via an ethernet cable and the displayed data is collated hourly from the HMI. The complete R markdown code utilized in running the descriptive analysis on the raw turbine dataset is shown in Ref. [9]. Among the library packages used in this code are ‘ggplot2’ which was used for all the plots in this data article. The ‘psych’ library provided the descriptive statistics for each set of related parameters considered. In filtering the dataset to remove all ‘not available (na)’ values and all non-significant features, the ‘dplyr’ library was utilized. The ‘readxl’ library read in the excel spreadsheet, and the ‘writexl’ library were used to write data-frames into excel spreadsheets. From the descriptive analysis performed on the raw dataset obtained, a total of nineteen (19) features were deduced. Of all 19 related parameters considered, only 16 parameters had a correlation co-efficient greater than 0.5 with respect to the target variable (real power).
